# Genetic and epigenetic alterations induced by bisphenol A exposure during different periods of spermatogenesis: from spermatozoa to the progeny

**DOI:** 10.1038/s41598-019-54368-8

**Published:** 2019-12-02

**Authors:** Marta Lombó, Cristina Fernández-Díez, Silvia González-Rojo, María Paz Herráez

**Affiliations:** 1 Department of Molecular Biology, Faculty of Biology and Environmental Sciences, Universidad de León, Campus de Vegazana, León, 24071 Spain; 2 Instituto Ganadero de Motaña (IGM), Finca Marzanas-Grulleros Vega de Infanzones, León, 24346 Spain

**Keywords:** Chromatin remodelling, Double-strand DNA breaks

## Abstract

Exposure to bisphenol A (BPA) has been related to male reproductive disorders. Since this endocrine disruptor also displays genotoxic and epigenotoxic effects, it likely alters the spermatogenesis, a process in which both hormones and chromatin remodeling play crucial roles. The hypothesis of this work is that BPA impairs early embryo development by modifying the spermatic genetic and epigenetic information. Zebrafish males were exposed to 100 and 2000 μg/L BPA during early spermatogenesis and during the whole process. Genotoxic and epigenotoxic effects on spermatozoa (comet assay and immunocytochemistry) as well as progeny development (mortality, DNA repairing activity, apoptosis and epigenetic profile) were evaluated. Exposure to 100 µg/L BPA during mitosis slightly increased sperm chromatin fragmentation, enhancing DNA repairing activity in embryos. The rest of treatments promoted high levels of sperm DNA damage, triggering apoptosis in early embryo and severely impairing survival. Regarding epigenetics, histone acetylation (H3K9Ac and H3K27Ac) was similarly enhanced in spermatozoa and embryos from males exposed to all the treatments. Therefore, BPA male exposure jeopardizes embryonic survival and development due to the transmission of a paternal damaged genome and of a hyper-acetylated histone profile, both alterations depending on the dose of the toxicant and the temporal window of exposure.

## Introduction

Bisphenol A (BPA; 2,2-bis(4-hydroxyphenyl)propane] was firstly used to enhance the growth of cattle and poultry due to its estrogenic activity, but its actual future would be in epoxy resins and plastic industry^1^. Since 1957, when chemists at Bayer and General Electric discovered that once this compound polymerizes it produces a hard plastic (known as polycarbonate), it has become an essential component for the manufacturing of beverage and feeding bottles, electronic devices, thermal paper, medical instruments, dental materials, and so on^2,3^. Owing to its widespread use in domestic and industrial products, the presence of BPA is ubiquitous: it has been detected in air, soil, water and food, from where it is able to migrate to urine, saliva, skin, blood, breast milk and even, amniotic fluid^4,5^. Despite the high variation of BPA concentrations in the environment, sewage effluents and landfill leachates have turned aquatic systems into the most common source of contamination, BPA concentrations rating from nM concentrations in river water samples to mM concentrations in landfill leachates as reviewed by Crain and colleagues^6^. The continuous exposure to this toxicant has been related to health problems in animal models, wildlife and humans^7^.

As summarized by Almeida and colleagues^8^, BPA displays a wide range of effects including diabetes, cardiovascular diseases, abnormalities in behavior and immune function, breast cancer and infertility. The deleterious effect of BPA on male reproductive function may happen during embryonic, pubertal and/or adult life^9^. During these last two periods of life, spermatogonial stem cells multiply and differentiate into mature spermatozoa, process known as spermatogenesis, which takes place in the extremely regulated microenvironment of the testis^4^. Although the gonadotropins LH (luteinizing hormone) and FSH (follicle stimulating hormone) are the most important hormones in charge of the regulation of testicular physiology, both thyroid and steroid hormones (estrogens and androgens) also play a crucial role on fish spermatogenesis^10,11^. Mackay and colleagues^7^ reviewed that BPA is an endocrine disruptor able to bind not only to estrogen receptors but also to androgen, thyroid hormone and estrogen-related receptors, so it likely interferes in any step of sperm cell formation^3^. In fact, BPA has been reported to induce restructuring of rat blood-testis barrier^12^ and to reduce sperm count and quality in many different species^13^.

Although it is widely known that BPA affects male reproduction owing to its endocrine disruptive capacity^13–15^, little is known about the mechanisms of inheritance to subsequent generations non-exposed to the toxicants. In that regard, both genotoxic and epigenotoxic effects on the germline cells might well lie behind the paternal transmission of the toxic effects to the progeny that have been previously reported^16^.

As far as the genotoxic effects are concerned, exposure to BPA has been described to cause meiotic arrest, to induce meiotic aneuploidy and chromosome aberrations, and to inhibit meiotic double strand breaks (DSBs) repair^17–19^. Moreover, adult male exposure to BPA has been reported to induce persistent DSBs in pachytene spermatocytes and to disrupt meiotic progression in rat and in zebrafish^20,21^. Despite the alterations occurred in the male germline, zygote still has the chance to manage this information and to mend some errors which are presumably inconsistent with proper embryo development. Even though maternal repairing machinery is essential to fix sperm DNA damage^22^, embryos also develop strategies to face unrepaired damage: they activate different pathways for detecting damaged DNA^23,24^ and, eventually, they decide whether fixing or tolerating the harm to keep on developing (if the damage is moderate)^25^ or inducing apoptotic activity and senescence (when DNA is harshly damaged)^23,26,27^.

Historically, spermatozoa have been considered as mere vectors, whose function was limited to deliver the haploid paternal genome to an oocyte during fertilization. Nowadays, much more attention has been paid to the role of paternal contribution on embryo development^28–30^. Albeit very specialized, sperm cells still handle much information that goes beyond nuclear DNA transmitted to the oocyte. The epigenetic landscape of spermatozoa is also transmitted and, therefore, it may have an impact on offspring health^28,31,32^. Throughout the spermatogenesis, the different cell populations suffer an intense remodelling of the epigenetic information, which involves inheritable modifications altering gene expression that do not change primary DNA sequence, such as DNA methylation, post-translational histone modifications as well as coding and non-coding RNAs^28,33^. Therein lies another important target of BPA, whose epigenetic toxicity has already been associated with reproductive disorders^34^. Concerning DNA methylation, BPA has been reported to cause both global hypomethylation in human spermatozoa^35^ and zebrafish testis^36^ and hypermethylation in mouse spermatocytes^37^ Changes in histone acetylation triggered by BPA are also dependent of doses and species: long-term exposure to a low dose of BPA led to a decrease in histone acetylation in rat testes^38^, whereas exposure to high doses of BPA induced an increase in histone acetylation in zebrafish testes^21^. Regarding epigenetic aberrations, embryos at early stages of development undergo a reprogramming of epigenetic marks, so they have the potential to erase and correct most epimutations carried by the spermatozoa^9,16^. Nevertheless, some environmentally-induced epigenetic defects can be inherited through paternal via by subsequent generations when these changes occur in elements escaping from remodelling, such as imprinted genes, or when the enzymes in charge of the epigenetic reprogramming are affected^39–41^.

Taking all these data into account, the aim of this work is to determine whether paternal BPA exposure during different periods of the spermatogenesis- when unique molecular modifications are being established- is able to alter the information contained in the spermatozoa, thus jeopardizing embryo development. For this purpose, we have used zebrafish (*Danio rerio*) as model species since its spermatogenesis has been deeply described^42^ and its early development is easy to monitor due to its external fertilization and the transparency of embryos^43^.

## Results

### Spermatozoa DNA fragmentation

**Figure 1:**
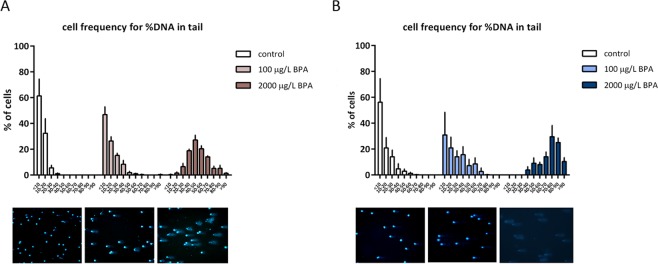
Percentage of spermatozoa in each rate of tail DNA in: (**A**) males exposed to BPA during mitotic phases and (**B**) during the whole spermatogenesis. Bars represent percentage of 4 pools of sperm cells from 4 males per treatment (n = 4).

The evaluation of DNA integrity in sperm cells revealed an increase in the number of cells with fragmented DNA displaying a different pattern of distribution among treatments, which depends not only on the BPA dose but also on the time of exposure (Fig. 1A,B). Regarding short exposure, around 46% of sperm cells from males treated with 100 µg/L BPA showed less than 10% of DNA fragmentation, whereas 27% of cells from males exposed to 2000 µg/L BPA had between 40 and 50% of their DNA fragmented (Fig. 1A). As for long exposure to 100 µg/L BPA, there was a decrease in the percentage of low-damaged sperm cells: 30% had less than 10% of fragmented DNA, whereas all the spermatozoa from males exposed to 2000 µg/L BPA exhibited DNA fragmentation higher than 40%; in fact, 29% of these cells had between 70 and 80% of fragmented DNA (Fig. 1B).

### Sperm epigenetics

**Figure 2:**
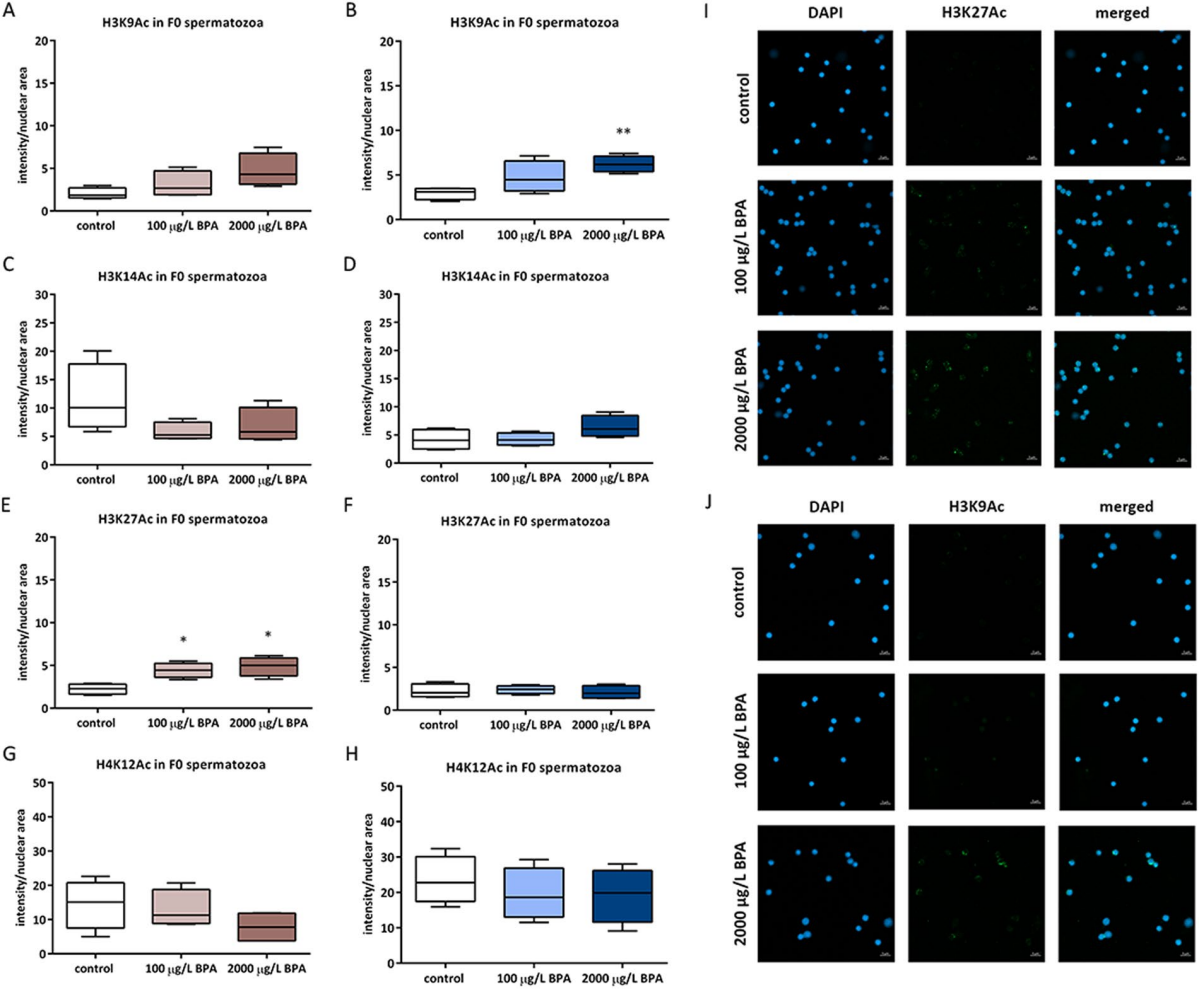
Quantification of histone acetylation marks: H3K9 (**A**,**B**), H3K14 (C and D), H3K27 (**E,F**) and H4K12 (**G,H**) in spermatozoa from males exposed to BPA during mitotic phase as well as the whole spermatogenesis, respectively. Boxes represent nuclear intensity of Alexa Fluor 488 in spermatozoa from 4 males per treatment (n = 4). Asterisks indicate significant differences (*p < 0.05; **p < 0.01) when comparing to control group. Representative images of histone acetylation at 100X (scale bar = 5 µm) appeared in: (**I**) H3K27Ac in spermatozoa from males exposed to BPA during mitotic phase and (**J**) H3K9Ac in spermatozoa from males exposed to BPA during all the spermatogenesis.

The assessment of epigenetic landscape of spermatozoa revealed that methylation of spermatozoa was not modified after exposing males to BPA (Supplemental Material, Fig. S2A, B) but two different acetylation marks did change after BPA exposure. Short exposure to both doses of BPA caused an increase in H3K27Ac and long exposure to the highest dose of BPA remarkably increased the acetylation of H3K9Ac (Fig. 2).

### Embryo mortality

**Figure 3:**
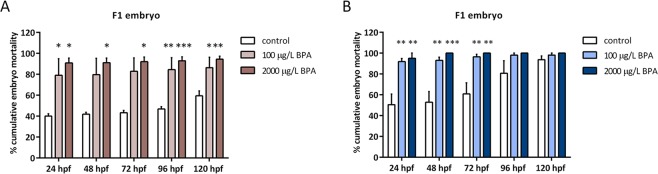
Percentage of embryo mortality throughout 120 hours of embryo development in: embryo obtained from males exposed to ethanol and BPA during mitotic phase of spermatogenesis (**A**) and males exposed during the whole spermatogenesis (**B**). Bars represent 4 batches of control and treated embryos (n = 4). Asterisks indicate significant differences (**p < 0.01; ***p < 0.001) when comparing to control group.

The mortality of embryos from control and males exposed to BPA during 2 and 3 weeks was evaluated from the first 24 hpf to 120 hpf. The results, indicated as percentage of cumulative mortality, showed a high increase in batches obtained from BPA-exposed males. Particularly, short exposure to 2000 µg/L BPA greatly increased F1 mortality but it never exceeded 94% (Fig. 3A). However, long exposure to the highest dose of BPA, led to 100% of embryonic mortality already at 48 hpf (Fig. 3B).

### Embryonic DNA repairing activity

**Figure 4:**
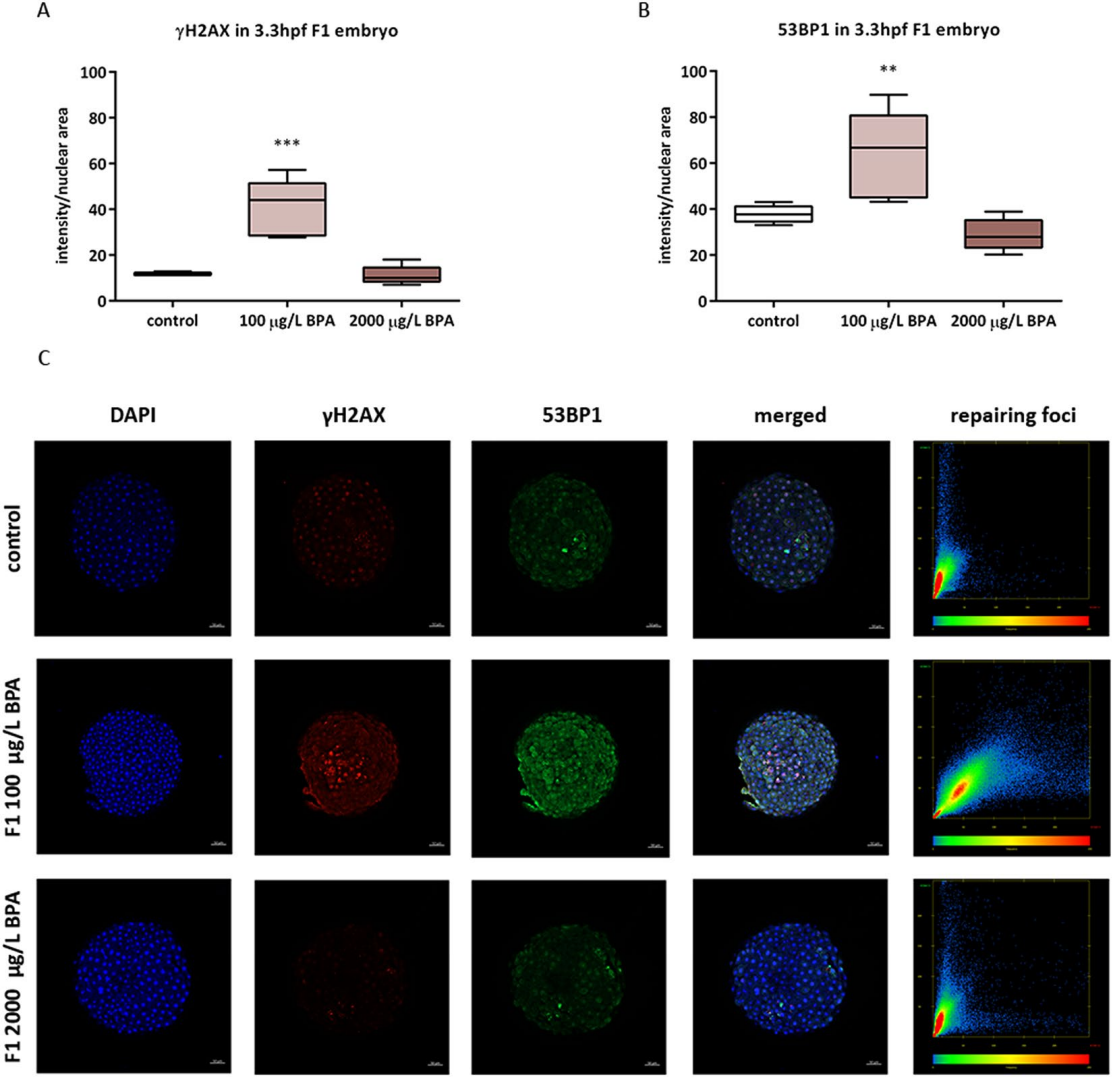
Quantification of γH2AX (**A**) and 53BP1 (**B**) in F1 embryos from males exposed to BPA during mitotic phase. Boxes represent median of around 200 cell of 5 embryos per treatment (n = 5). Asterisks indicate significant differences (**p < 0.01; ***p < 0.001) when comparing to control group. Representative images of 3.3hpf-embryo at 5X (scale bar = 50 µm) appeared in (**C**). Channels were both separated in blue (DAPI), red (Alexa Fluor 568) and green (Alexa Fluor 488) and merged. The graphic represents the co-localization of red channel (γH2AX) in X–axis and green channel (53BP1) in Y-axis.

**Figure 5:**
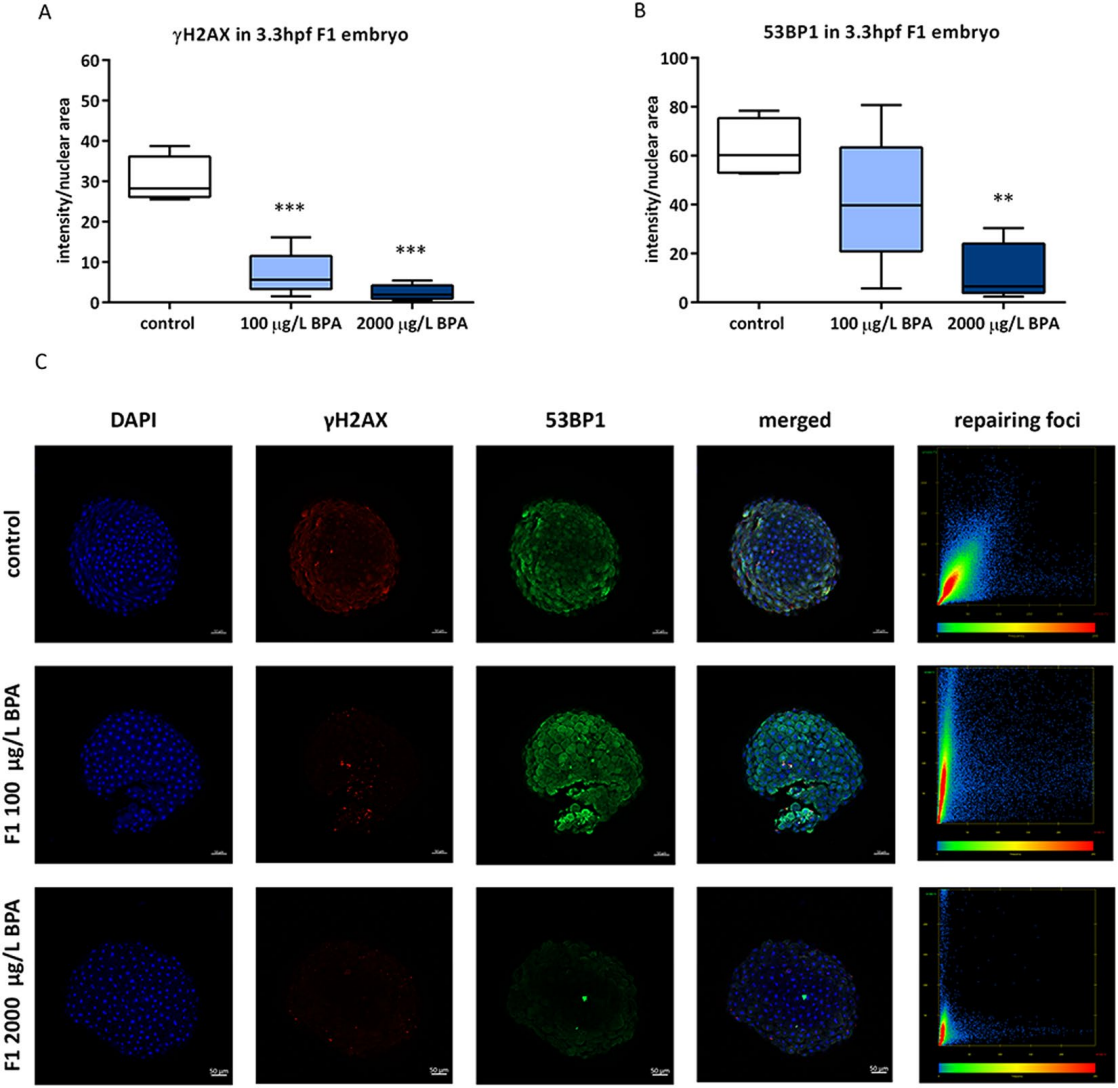
Quantification of γH2AX (**A**) and 53BP1 (**B**) in F1 embryo from males exposed to BPA during all the spermatogenesis. Boxes represent median of around 200 cell of 5 batches per treatment and timing (n = 5). Asterisks indicate significant differences (**p < 0.01; ***p < 0.001) when comparing to control group. Representative images of 3.3hpf-embryo at 5X (scale bar = 50 µm) appeared in (**C**). Channels were both separated in blue (DAPI), red (Alexa Fluor 568) and green (Alexa Fluor 488) and merged. The graphic represents the co-localization of red channel (γH2AX) in X–axis and green channel (53BP1) in Y-axis.

The activation of DNA damage signalling in the embryos was assessed at 3.3 hpf via two markers: γH2AX and 53BP1, whose combination in the nuclei gives rise to the repairing foci. The progeny from males exposed to 100 µg/L BPA during mitotic phase displayed an increase in these two proteins (Fig. 4A,B) and a high level of their co-localization (Fig. 4C). None of these results were reproduced by the offspring of males treated with 2000 µg/L BPA. Concerning the progeny from males exposed during the whole spermatogenesis, there was a decrease in γH2AX in embryos from males treated with the two doses of BPA (Fig. 5A) and a reduction in 53BP1 of embryos from males exposed to 2000 µg/L BPA (Fig. 5B). The study of co-localization did not indicate any formation of repairing foci (Fig. 5C).

### Apoptotic activity

**Figure 6:**
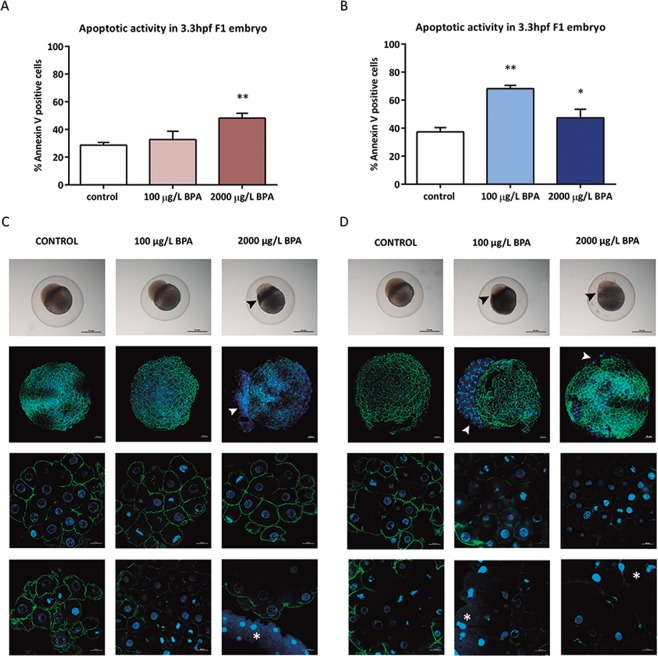
Assessment of apoptotic activity in: embryos from control males and males exposed to BPA during 2 weeks (**A**) and from males exposed to BPA during 3 weeks. Bars represents mean of 20,000 cells (20 embryos at 3.3hpf) of 4 different embryo batches per treatment (n = 4) (*p < 0.05; **p < 0.01). Representative images of 3.3hpf-embryo at light and confocal microscopy appeared in (**C,D**). For fluorescence images snaps were taken at 5X (scale bar = 50 µm) and 40X (scale bar = 50 µm) in both channels: blue for DAPI and green for Alexa Fluor 488 Phalloidin. Arrows point to hypertrophied YSL whereas asterisks highlight the presence of nuclei within the syncytium.

The results revealed that the apoptotic activity of F1 embryos obtained from males treated with 2000 µg/L BPA during early spermatogenesis reached 49.6%, being significantly higher than in control batches (Fig. 6A) and it also increased in F1 embryos up to 68.2% and 51.9% when males were exposed to 100 µg/L and 2000 µg/L BPA, respectively, during the whole spermatogenesis (Fig. 6B).

### Yolk syncytial layer formation

Immunostaining with phalloidin, which clearly showed the cell cortex in the blastomeres, showed that embryos from treatments without the ability to form repairing foci in which the apoptosis was enhanced, displayed an abnormal development of yolk syncytial layers (YSL), which resulted hypertrophied (Fig. 6C,D). This syncytium consists of an extra-embryonic tissue formed by the tenth cell division (during the blastula stage) in all teleost fishes.

### Embryonic epigenetics

#### Methylation

The analysis of global DNA methylation (5mC) at 3.3 hpf did not reveal any difference among embryos from control and males exposed to BPA during any period of spermatogenesis. The expression analysis of several DNA-methyltransferases (*dnmts*) at this stage of development did not show any alterations either (Supplemental Material, Fig. S2).

#### Acetylation

**Figure 7:**
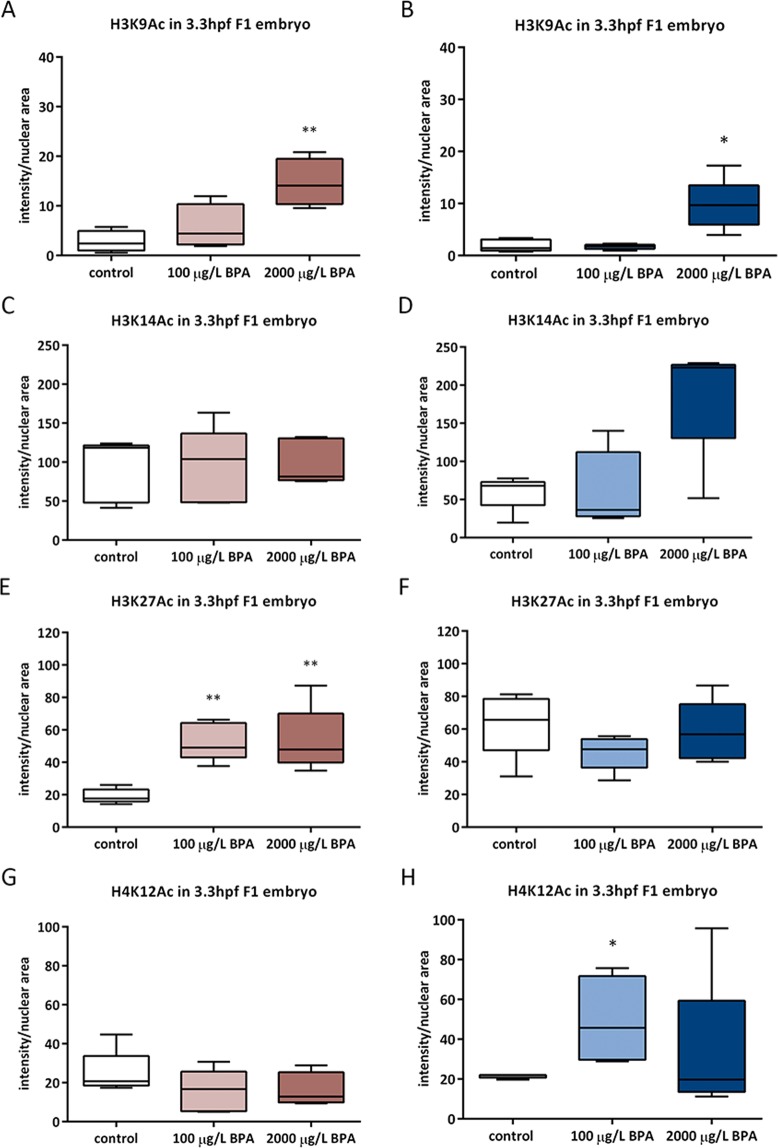
Analysis of several histone marks prone to acetylation: H3K9 (**A,B**), H3K14 (**C,D**), H3K27 (**E,F**) and H4K12 (**G,H**) in embryo at 3.3 hpf from males exposed to BPA during mitotic phase as well as the whole spermatogenesis, respectively. Boxes represent nuclear intensity of around 200 cell of 5 batches per treatment and timing (n = 5). Asterisks indicate significant differences (*p < 0.05; **p < 0.01) when comparing to control group.

The evaluation of histone acetylation in embryos at 3.3 hpf showed modifications in several marks. There was an increase in acetylation of lysine 9 in histone 3 (H3K9Ac) of embryos from males exposed to 2000 µg/L BPA during both periods of spermatogenesis (Fig. 7A,B). Furthermore, acetylation of lysine 27 in histone 3 (H3K27Ac) was also increased in the offspring from males exposed to BPA during mitotic phase (Fig. 7E) and the rise in acetylation of lysine 12 in histone 4 (H4K12Ac) was observed in embryos from males exposed to 100 µg/L BPA during the whole spermatogenesis (Fig. 7H).

**Figure 8:**
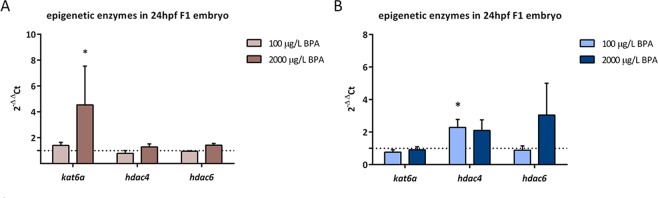
Evaluation of epigenetic enzymes expression in: 24hpf-embryo from males exposed to BPA only during mitotic phase (**A**) and also meiotic phase and spermiogenesis **(B**). Bars represent expression levels relative to *actb2*, which were calculated using 2^−ΔΔCt^ method of three independent experiments (n = 3). Asterisks indicate significant differences (*p < 0.05) when comparing to control group (dashed line).

Additionally, expression of three enzymes involved in histone acetylation was evaluated at 24 hpf: *kat6a* (histone acetyltransferase), *hdac4* and *hdac6* (histone deacetylases). Results revealed an overexpression of *kat6a* in embryos from males treated 2 weeks with 2000 µg/L BPA (Fig. 8A). In contrast, exposure to 100 µg/L BPA during 3 weeks led to an upregulation of *hdac4* (Fig. 8B).

## Discussion

Several studies have reported that exposure to environmental pollutants may lead to alterations of the information contained in the spermatozoa, thus endangering the health of subsequent generations^44,45^. It is also known that the risk of disease as well as its transmission depends on the susceptibility window during which the exposure to the toxicant takes place^9^. Spermatozoa are exceptionally specialized cells formed through a differentiation process which involves mitotic expansion of spermatogonia, meiotic phase when primary and secondary spermatocytes are generated and, eventually, the spermiogenesis, a process in which haploid spermatids turn into motile and flagellated spermatozoa^10^. Understandably, different molecular mechanisms lie behind each of these mentioned steps including among others chromatin compaction, which implies transient DNA strand breaks^46^ and epigenetic events in DNA and its associated histones^47^. Our findings clearly demonstrate that both genetic and epigenetic information of sperm cells, as well as F1 early embryo, are decidedly altered after paternal exposure to BPA during two different periods of spermatogenesis: one involving only mitotic phase and another one affecting the whole process of spermatogenesis. And, what is more, most of these effects vary in a time-dependent manner.

BPA exposure during mitotic phase triggered an increase in the percentage of embryo mortality, reaching 94% in the progeny from males exposed to the highest dose. After this period of exposure, half of the spermatozoa from males treated with 100 µg/L BPA showed a slight decrease in DNA integrity (around 46% of sperm cells from males treated with 100 µg/L BPA showed less than 10% of DNA fragmentation). In contrast, in males exposed to 2000 µg/L BPA sperm DNA fragmentation is much higher, 27% of cells having between 40 and 50% of fragmented DNA. Single strand breaks in sperm are further transformed in double strand breaks in the embryos during replication^48^. DSBs induce the recruitment of many DNA damage response (DDR) proteins such as the sensor γH2AX (phosphorylated H2AX) and the mediator 53BP1 (p53-binding protein)^49,50^. This is in accordance with the ability of those embryos to initiate DNA repair: progeny from males exposed to 100 µg/L BPA showed higher levels of γH2AX and 53BP1, both proteins co-localizing for repairing foci generation. Similar responses to deal with DNA fragmentation were observed by our group in 3.3hpf-embryo obtained from sperm treated with UV during 30 and 40 s^25^. However, the scenario observed in embryos from males exposed to the highest dose of BPA was completely different: due to the great deal of sperm DNA fragmentation, no repairing potential was observed in the progeny.

The effects are more severe after BPA treatment during the whole spermatogenesis, embryo mortality overtaking 90% in batches from males exposed to 100 µg/L BPA and reaching 100% two days after fertilization when fathers were exposed to 2000 µg/L BPA. This considerable boost in embryonic death is again closely related to the sperm DNA fragmentation, which highly increases in males treated with the toxicant during the whole spermatogenesis. After the treatment with 100 µg/L BPA only 30% of spermatozoa had less than 10% of fragmented DNA, whereas all the cells from males exposed to 2000 µg/L BPA exhibited DNA fragmentation higher than 40%; 29% of them having between 70 and 80% of fragmented DNA. Both, meiosis and spermiogenesis, imply DNA strand breaks allowing the chromosome crosslinks during pachytene and the chromatin compaction during spermiogenesis^51^, therefore they arise as sensitive phases to BPA genotoxic damage. BPA has been already described to increase meiotic DNA strand breaks in pachytene spermatocytes, triggering meiotic disruption in rat^20^. The induced DNA damage will be transmitted unrepaired by the sperm to the zygote, as it has been reported in mammal and fish studies^25,52^. Analysis of the repairing ability showed a significant decrease in γH2AX on embryos from males treated with both doses of BPA and also in 53BP1 on embryos from males exposed to the highest dose of BPA. Phosphorylation of H2AX together with 53BP1 have been considered as biomarkers for DDR^53^, so our results showed that F1 embryos from males exposed to BPA during all the spermatogenesis were not able to face genomic fragmentation of the sperm.

Bearing in mind that paternal DNA damage is basically repaired in the zygote during early development^22,54^, and that zebrafish 3.3 hpf-embryos obtained with a certain degree of sperm DNA damage show an intense repairing activity^25^, the failure of repairing activity observed in the offspring likely leads to the activation of apoptosis^55^. Indeed, this mechanism represents a cell fate described to face unrepaired DNA fragmentation^24^. DNA repair was only accomplished in batches from males treated with the lowest doses of BPA during the mitotic phase, which showed basal levels of apoptosis. In contrast, the rest of the treatments causing high levels of sperm DNA damage led to embryonic inability to activate neither DNA damage signaling nor DNA repair, promoting an increased apoptotic activity. A similar apoptotic pattern was reported by our group in 8hpf-embryo obtained from sperm with 40% DNA in tail, who also showed lower levels of γH2AX and, therefore, an absence of DNA repairing ability^25^ when the damage overcame the embryo repair capacity. Interestingly, Kao and colleagues^56^ reported that repair factors, such as 53BP1, are stabilized by a histone deacetylase (HDAC4), thus silencing of HDAC4 results in decreased 53BP1 protein level. Hence, the maintenance of 53BP1 levels in the progeny of males exposed to 100 µg/L BPA, in spite of their incapacity to form repairing foci, may be associated with the overexpression of *hdac4* detected in these embryos.

Another mechanism involved in the DDR is orchestrated by Rho GTPases, since they control the accessibility of the chromatin for DNA repair and they allow the entrance of DDR factors to the nucleus by modifying actin cytoskeleton^57^. Even though the analysis of actin cytoskeleton with phalloidin did not reveal any evident alteration of actin distribution in the blastomeres among treatments, it did allow the observation of dramatic changes in the formation of an extraembryonic tissue, known as yolk syncytial layer (YLS). The formation of YSL occurs at blastula stage due to the absence of cytokinesis, a process regulated by the same small GTPases of Rho family^58^ which is dependent of the reorganization of the actin cytoskeleton. YSL is crucial for organizing both endoderm and mesoderm, cardiac progenitors formation and regulation of larval metabolism^59^. Confocal imaging showed that embryos who were not able to repair DNA displayed hypertrophic YLS, similar to that produced when Rock (protein which binds to Rho GTPases) was inhibited^58^, suggesting that paternal BPA exposure causes embryonic dysregulation of this signaling pathway, which could also have an impact on DDR.

Besides genotoxic effects of BPA, epigenetic modifications triggered by this toxicant must also be considered. The first study concerning epigenotoxicity of BPA revealed that maternal exposure in mice induced a change in F1 coat coloration pattern due to a reduction in methylation of IAP (Intracisternal A-Particle) of *Agouti* gene promoter^60^. From this moment onwards, many investigations have confirmed that BPA is able to modify DNA methylation in both female^61,62^ and male germline^21,35,36^. However, in our previous study of paternal BPA exposure, during mitotic spermatogenesis, no alterations in global DNA methylation of sperm or testicular cells were found^45^. Neither did DNA methylation in the progeny from males exposed to BPA during different windows change, nor the expression of DNA-methyltransferases. In contrast to DNA methylation, BPA effects on histone acetylation have barely been studied. Recent results from our group have demonstrated that zebrafish male exposure to the same doses of BPA (100 and 2000 µg/L BPA) triggered an increase in H3K9Ac, H3K14Ac and H4K12Ac in testicular cells^21^. However, Chen and colleagues^38^ reported a decrease in H3K9Ac, H3K27Ac and H4K12Ac in rat testes after long term exposure to a low dose of BPA. In this work, the evaluation of histone modifications in spermatozoa showed an increase in H3K27Ac and H3K9Ac after two and three weeks of BPA exposure, respectively. Moreover, this marks were also more acetylated in the progeny of these males: H3K9Ac and H3K27Ac were increased in embryo from males exposed to BPA during mitotic phase of spermatogenesis and H3K9Ac and H4K12Ac rose in embryo from males treated with BPA during the whole spermatogenesis. As a result of a continuous remodeling process, sperm epigenome becomes very susceptible to environmental factors and, when disrupted, may result in male infertility as well as in abnormal embryo development^28,31^. Specifically, histone modifications in sperm are of utmost importance for next generation development^9^. In fact, alterations of histone epigenetic marks in mature spermatozoa have been associated with abnormal embryo gene expression and phenotype^63–65^. Our results showed that changes in sperm histone acetylation entailed similar changes in early embryos. The paternal transmission of these epigenetic alterations might be explained by changes in the expression of a histone acetyltransferase *(kat6a)*.

In this study we have demonstrated that effects of male BPA exposure on sperm information and the consequences for the next generation strongly depend on the doses and on the period of spermatogenesis affected. Hence, exposure during the whole spermatogenesis leads to more sperm DNA damage than that caused by exposure only during mitotic phase. Due to this excessive fragmentation, the embryo capacity to activate the DNA repairing machinery is exceeded and, therefore, they initiate an apoptotic process. Our findings showed that paternal BPA exposure, even to 100 µg/L only during mitotic period, triggered modifications in sperm histone acetylation and, what is more, these epigenetic alterations were transmitted to the next generation, thus jeopardizing early embryo development. Hence, this work highlights the potential of environmentally-induced alterations to be inherited by future generations.

## Methods

### Ethics statement

This work is included in a project from the Spanish Ministry of Economy and Competitiveness (Project AGL2014-53167-C3-3-R) specifically approved by the University of León Bioethical Committee as well as by the competent body of Junta de Castilla y León (project number: ULE009-2016). All the animals were manipulated in accordance with the Guidelines of the European Union Council (86/609/EU, modified by 2010/62/EU), following Spanish regulations (RD 1201/2005, abrogated by RD 53/ 2013) for the use of laboratory animals.

### Zebrafish maintenance

8-month-old zebrafish, AB strain (wildtype), were maintained in 2.5 L aquaria (ZebTEC, Tecniplast System) with a recirculating water system (pH 7.0–7.5, 30 mg/L Instant Ocean, at 27–29 °C, 14:10 light-dark cycle). Animals were fed twice a day with dry food (Special Diets Services).

### BPA paternal exposure

The adult zebrafish males (4 fishes per replicate) were exposed to vehicle (ethanol 0.014% (v/v)) and two doses of BPA previously tested by our group^45^: 100 and 2000 μg/L (0.44 μM and 8.8 μM, respectively) in 1.5 L of zebrafish water. More than 20 replicates per treatment were performed to obtain all the required samples. The tolerable daily intake (TDI) of BPA has been established by the EFSA in 4 µg/kg weight/day for humans^64^. In comparison with these values, the doses used in this work are high. However, taking into account the health policy and the environmental concentrations, 100 μg/L BPA was established as the total allowed concentration in drinking water by the Environmental Protection Agency (EPA) in 2008^66^, whereas 2000 μg/L BPA fits in the normal levels of BPA on waste landfill leachates^67^. Moreover, the EPA recommends setting the highest dose level at the maximum tolerated dose (MTD). Based on the guidelines of aquatic tests, MTC is approximately one third of the fish acute 96-h LC_50_ (the dose inducing 50% mortality at 96 hpf). In zebrafish, this dose has been established in 12 mg/L^68^, so we are still below half the highest recommended dose, that would be 4 mg/L. Given that zebrafish spermatogenesis has been estimated to last around 21 days^10^ and meiotic and spermiogenic phases to last 6 days^42^, fishes were subjected to BPA exposure during two different periods: one affecting mitotic phase −2 weeks, short exposure- and another one affecting also meiosis and spermiogenesis −3 weeks, long exposure- (Supplemental Material, Fig. S1).

### Comet assay in spermatozoa

4 samples of pooled sperm from 4 control or 4 BPA-exposed males each, were obtained by abdominal massage and diluted in PBS to a final concentration of around 10^6^ cells/mL. In order to assess DNA fragmentation, Single Cell Gel Electrophoresis (SCGE) was performed as described by Fernández-Díez and colleagues^25^. Sperm cells were mixed with 0.5% low melting point agarose and 75 μL of cell suspension were spread out on ATE ([3-aminopropyl]trimethoxysilane) coated slides with a glass coverslip. Once they have solidified, coverslips were removed and slides were incubated 1 h at 4 °C in lysis solution (100 mM EDTA-Na_2_, 2.5 M NaCl, 10 mM Tris-HCl and 1% Triton X-100, pH 10). Then, they were placed into an electrophoresis buffer (1 mM EDTA-Na_2_, 0.3 M NaOH, pH 13) for 20 min to promote the DNA unwind followed by 20 min of electrophoresis (25 V, 280–350 mA). The slides were washed using a neutralizing solution (0.4 M Tris-HCl, pH 7.5). Cells were fixed with methanol and nuclei were stained with 0.5 μg/mL 4′, 6-diamidino-2-phenylindole (DAPI). 50 images per replicate (4 in each treatment) were analysed with CaspLab software (1.2.3beta2; http://www.casp.of.pl.), the percentage of tail DNA (% DNAt) being used as a measure of DNA fragmentation.

### Immunostaining of epigenetic marks in spermatozoa

Sperm from control and BPA-exposed males was collected as indicated above. Samples were fixed with 4% (wt/vol) paraformaldehyde for 20 min at room temperature and washed twice with bi-distilled water. 25 µl of spermatozoa from each individual male (4 males per treatment and period of exposure) were spread out on ATE coated slides at 37 °C overnight. Following steps were done using the method described by González-Rojo and co-workers^69^. Primary antibodies and working dilutions are indicated in Supplemental Material, Table S1. Slides were mounted with ProLong Gold Antifade Mountant (Thermo Scientific) and observed under confocal microscope LSM 800 (Zeiss). Relative quantity of each mark was quantified in around 200 cells per sample using ImageJ software.

### Embryo collection and mortality

So as to obtain the embryos, control and exposed males were mated with non-treated females according to a *sex ratio* 1:2. Embryos were immediately rinsed 2 min in 0.5% (vol/vol) bleach and 10 s in 70% (vol/vol) ethanol. Then, they were transferred to egg water containing 0.038 mM CaCO_3_, 0.446 mM NaHCO_3_, 1.025 mM sea salt and 0.005% (vol/vol) methylene blue and kept at 28 °C until further analysis. Embryo mortality was evaluated from 24 hpf (hours post fertilization) each day until they reached 120 hpf.

### Gene expression analysis in embryo

Each RNA sample was extracted from 30 embryos per batch obtained from control and BPA-exposed males (3.3 hpf and 24 hpf) using Trizol reagent (Invitrogen, Spain) according to the manufacturer’s protocol. The concentration and purity (A260/A280 = 2.2–2) of RNA samples was measured using the NanoDrop ND-1000 UV-Vis Spectrophotometer (Thermo Scientific), whereas the integrity was assessed by electrophoresis on agarose gel. For cDNA syntheses and RT-qPCR, the same protocols and primer sequences for DNA methyltransferases (*dnmt1*, *dnmt3*, *dnmt5*, *dnmt8*), histone acetyltransferase (*kat6a*) and histone deacetylases (*hdac4* and *hdac6*) as those previously described by our group^70^ were used.

### Embryonic whole mount immunostaining

Embryos at 3.3 hpf, obtained from control and treated males, were fixed in 4% (wt/vol) paraformaldehyde overnight at 4 °C. After washing twice in PBS (8.37 mM Na_2_HPO_4_, 1.83 mM KH_2_PO_4_, 149.9 mM NaCl, pH 7.4) and removing both chorion and yolk sac, cells were permeabilised with methanol 2 hours at −20 °C. For 5mC and γH2AX/53BP1 analysis, an extra step of 2 h DNA denaturalization with 2 N HCl was performed. Then, all embryos were washed 3 times with TBS-T 1% (vol/vol) and transferred to blocking solution (3% (wt/vol) BSA in TBS-T 1%) for 2 h at room temperature. Embryos were incubated for 2 days at 4 °C in blocking solution with the primary antibodies described in Supplemental Material, Table S1. Next, they were rinsed in fluorescence-conjugated secondary antibodies (goat anti-mouse AlexaFluor 568 and goat anti-rabbit AlexaFluor 488 (Invitrogen)) at 4 °C overnight. For fluorescent labelling of actin, embryos were fixed as described above and directly rinsed in PBS-T 2% (vol/vol) for 2 h. Then, they were incubated with Alexa Fluor 488 Phalloidin for 1 h. Eventually, nuclei were stained with 180 μM DAPI for 8 min. Whole embryos were mounted with ProLong Gold Antifade Mountant (Thermo Scientific) and observed under confocal microscope LSM 800 (Zeiss) using ibidi. Relative quantity of each mark was quantified using ImageJ software.

### Apoptotic activity

20 embryos per batch from control and BPA-exposed males were collected at 3.3 hpf and they were dechorionized. Blastomeres were washed with PBS, transferred to a tube and centrifuged at 1000*xg* 5 min at 4°c. Pellets were re-suspended in annexin buffer to perform the indicated protocol (FITC annexin V Apoptosis Detection Kit (Molecular Probes by Life Technologies)). Cells (20,000 events per sample) were analyzed by flow cytometry (MACSQuant Analyzer 10) and apoptotic activity was established as percentage of annexin V-positive cells.

### Statistical analyses

Statistical analyses were performed with SPSS version 24.0 (IBM). The normality of the data was tested by the Shapiro-Wilk’s test. For non-parametric data, a Kruskal-Wallis test was performed using Dunn’s post hoc test (p < 0.05). For parametric data, an analysis of variance (ANOVA) with a DMS post hoc test was carried out (p < 0.05). All data in bars are represented as mean ± SEM, whereas boxes represent median ± maximum and minimum.

## Supplementary information

Supplemental material
