# Supplementary material for: Genetic and epigenetic alterations induced by bisphenol A exposure during different periods of spermatogenesis: from spermatozoa to the progeny
Source: Sci Rep. 2019 Dec 2;9:18029. doi: 10.1038/s41598-019-54368-8 (PMC6889327; doi:10.1038/s41598-019-54368-8)
Supplement: Supplementary file 1 — Supplemental material [file 41598_2019_54368_MOESM1_ESM.docx]

**SUPPLEMENTAL MATERIAL**

**TITLE:** Genetic and epigenetic alterations induced by bisphenol A exposure during different periods of spermatogenesis: from spermatozoa to the progeny

**AUTHORS:** Marta Lombó, Cristina Fernández-Díez, Silvia González-Rojo, María Paz Herráez

**TABLES**

| Target | Antibody type | | Working dilution in sperm | Working dilution in embryos | Manufacturer | Product Code |
| --- | --- | --- | --- | --- | --- | --- |
| 5mC | | Mouse monoclonal | 1/200 | 1/500 | Abcam | ab10805 |
| H3K9Ac | | Rabbit monoclonal | 1/200 | 1/500 | Cell signaling | C5B11 |
| H3K14Ac | | Rabbit polyclonal | 1/100 | 1/200 | Abcam | ab82501 |
| H3K27Ac | | Rabbit polyclonal | 1/200 | 1/500 | Abcam | ab4729 |
| H4K12Ac | | Rabbit polyclonal | 1/200 | 1/500 | Abcam | ab46983 |
| γH2AX | | Mouse monoclonal | - | 1/50 | Abcam | ab26350 |
| 53BP1 | | Rabbit polyclonal | - | 1/100 | Abcam | ab36823 |
| Alexa Fluor 488 Phalloidin | | - | - | 1/200 | Molecular probes | A12379 |

**Table S1 І** List of antibodies used for the assessment of epigenetic marks (DNA methylation and histone acetylation) and of DNA repairing foci.


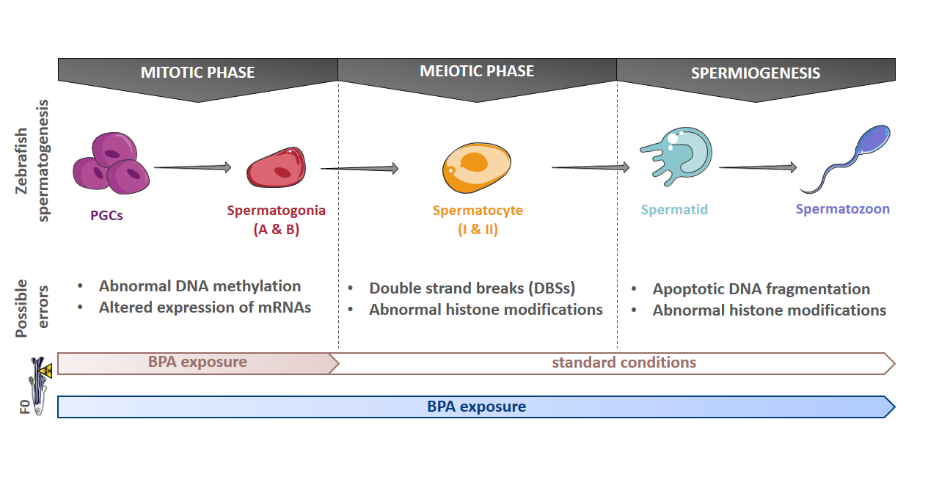


**Figure S1 І** Scheme of experimental design, representing two periods of BPA male exposure: one only affecting the mitotic phase of spermatogenesis (two weeks of treatment) and another one affecting both mitotic and meiotic phase as well as spermiogenesis (three weeks of treatment). Possible errors occurring in the different phases of spermatogenesis have been reported by Dada and colleagues (2012). For the design of this figure the cell drawings have been taken from <https://smart.servier.com/>. The colour of these images has been modified by the authors according to the license provided by the website <https://creativecommons.org/licenses/by/3.0/>.


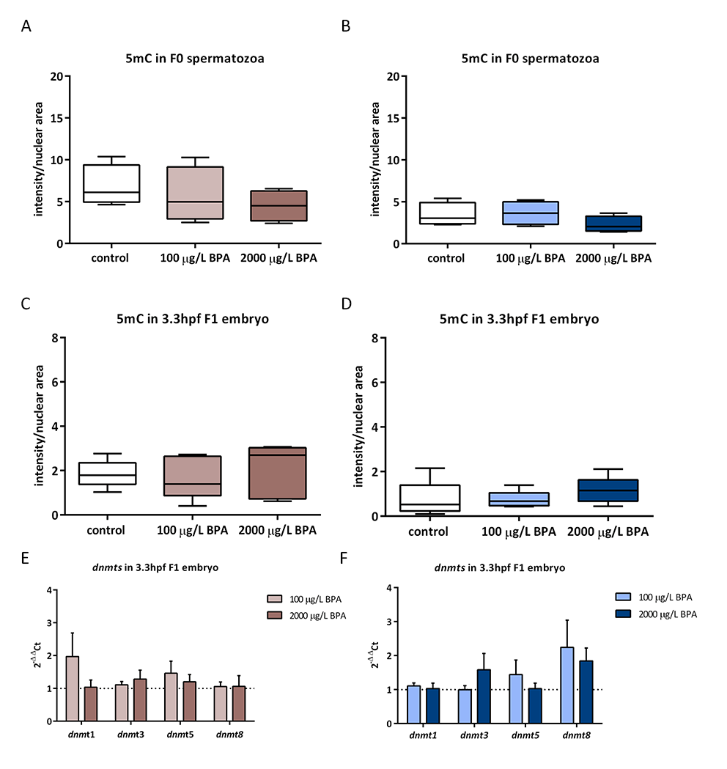


**Figure S2 І Analysis of global DNA methylation.** Relative measure of 5mC in spermatozoa from males exposed to BPA during mitosis (A) and all the spermatogenesis (B). Relative measure of 5mC in spermatozoa analysed by whole-mount immunostaining in 3.3hpf-embryo from males exposed to BPA only during mitotic phase (C) and also meiotic phase and spermiogenesis (D). Boxes represent nuclear intensity of around 200 cell of 5 embryos per treatment (n=5). Relative expression of DNA-methyltransferases in 3.3hpf-embryo from males exposed to BPA only during mitotic phase (E) and also meiotic phase and spermiogenesis (F). Bars represent expression levels relative to *18S rRNA*, which were calculated using 2^-ΔΔCt^ method of three independent experiments (n=3).
